# The Medical Needs of the Army and of the Hospitals

**Published:** 1915-09-18

**Authors:** 


### The Hospital

The Workers' Newspaper of Administrative Medicine and Institutional Life, Administration, National Insurance and Health.

No. 1527, Vol. LVIII. SATURDAY, SEPTEMBER 18, 1915. PRICE ONE PENNY.

### THE MEDICAL NEEDS OF THE ARMY AND OF THE HOSPITALS.

Whoever else may escape the urgent problems raised by the war, no such attitude of mind is possible either to the hospitals or the medical profession. The Army continues its demand for more doctors and the hospitals have increasing difficulty in securing resident officers; and neither in the one case nor in the other does there seem any prospect of a relaxation of the strain. Manifestly there is some measure of opposition between the two claims, and it is in the highest degree desirable that this opposition shall be reduced to the lowest possible limit. A still more desirable end would be the substitution of co-operation for competition. The aim of every arrangement just now must be a satisfaction of the military needs with, at the same time, reasonable provision for the care of the inmates of the civil hospitals. Some steps have recently been taken to this end in the scheme which permits newly-qualified practitioners to accept commissions, with a liability to be called to active service on short notice, while holding resident appointments in certain hospitals. This is so much to the good, but we believe something more may be done, and help towards the common purpose may be contributed from both sides.

In the first place we would suggest that the arrangement just indicated should receive a wide extension. There is no obvious reason why the proposal should be made to apply to a few hospitals only. One of the desired ends is that the Army shall have men at its discretion when the need for them arises, and this is satisfied wherever the hospital be situated. Secondly, it is obviously well that, except under some very urgent claim, the young man who has just acquired his diploma shall not be rushed into the considerable responsibilities of active service. A resident appointment, valuable from many points of view, is especially desirable for a man who has to undertake duties which involve the care and treatment of troops exposed to the risks and dangers of war. Why, then, should not the advantages of such appointments be made available on the widest possible scale? In other words, let all general hospitals be encouraged to offer resident appointments to young men who have recently qualified, who accept commissions, and who hold themselves at the absolute disposal of the authorities on short notice. In view of the urgency of the situation the terms of such appointments might be limited to three months, for, while this is but a brief period, it is far better than the complete absence of all independent hospital service. Such an arrangement would help to secure the volunteers for whom the Army continues to call, and it would make certain that such volunteers had had at least some measure of special practical training. It is becoming increasingly evident that men actually engaged in practice find it more and more difficult to respond to the invitation to give full-time service in the R.A.M.C. This means that the doctors which the Army needs must be mainly recruited from those who have recently qualified. There is a widespread belief that at the moment the special need of the authorities is a guarantee that the men will be forthcoming when they are wanted, rather than a desire for immediate personal service. This guarantee is not inconsistent with the holding of a resident appointment, provided that the holder accepts the conditions here suggested. With all this there would be combined some help for the civil hospitals. For manifestly a resident appointment which does not conflict with the patriotic claim is in a very different position from one which suggests an indisposition for Army duties. The hospitals, instead of appearing to be in conflict and competition with the military demand, would proclaim their recognition of the justice of this demand and their desire to contribute to its satisfaction.

There are, of course, two parties to the bargain here proposed, and the hospitals and hospital staffs must be willing to do their share. First of all is the necessity to be ready to accept resident officers who may at a few hours' notice be called away by a military summons; no doubt this means the risk of inconsiderable inconvenience, but the national cause claims a readiness to put up with inconvenience, and, as we have frequently urged, a visiting staff in such a time as the present ought willingly to share among themselves resident duties. Secondly, the hospitals ought to intimate very plainly that resident appointments will be given only to men who have accepted commissions or who are unfit, for physical or other reasons, for military duties. No one in civil life just now would employ a man-servant who might well be a soldier, and the hospitals ought to act on the same principle, and ought to let it be known that they are acting on this principle. Rightly or wrongly, it is believed among practitioners that while they are being invited to leave their practices and their families the hospitals are providing comfortable billets for younger and freer men, and some colour is lent to this belief by repeated advertisements which give no indication that men physically fit for service with the troops will not be accepted. Were some such qualification announced, the sense of injustice just alluded to would disappear. The hospitals would be known as active participators in the ambition to secure what the Army needs, and they would do this partly by refusing men who ought to be at the Front, and partly by offering special opportunities to those who have accepted commissions.

